# The effect of *Saliva officinalis* extract on the menopausal symptoms in postmenopausal women: An RCT

**DOI:** 10.18502/ijrm.v17i4.4555

**Published:** 2019-06-13

**Authors:** Fereshteh Dadfar, Kourosh Bamdad

**Affiliations:** Department of Biology, Payame Noor University, Tehran, Iran.

**Keywords:** *Plant*, * Sage*, * Menopause*, * Women*, * Post menopause*

## Abstract

**Background:**

The menopausal symptoms are the most common problems in postmenopausal women. Due to the side effects of hormone replacement therapy, the use of medicinal herbs has increased for the treatment of menopausal symptoms.

**Objective:**

The aim of this study was to evaluate the effect of *Saliva officinalis* on the decreasing of the severity of the menopausal symptoms in postmenopausal women.

**Materials and Methods:**

The study was performed on 30 postmenopausal women aged 46–58 yr referred to the healthcare center of Darab who experienced various degrees of postmenopausal symptoms. The severity of menopausal symptoms is recorded by a Menopause Rating Scale. Participants received a 100 mg capsule of sage extract daily for 4 wk. The severity of postmenopausal symptoms was compared before and after four weeks of the consumption of sage extract.

**Results:**

The results showed the severity of hot flashes, night sweats, panic, fatigue, and concentration had significant differences before and after the consumption of sage extract.

**Conclusion:**

It was concluded that *Saliva officinalis* were effective to change the severity of some of the menopausal symptoms in postmenopausal women.

## 1. Introduction

Menopause is the stage of life that all women experience in middle age. This phenomenon begins in the age range of 45 to 55 and is associated with a variety of physical and mental changes. At this time, the ovaries secrete less female hormones, especially estrogen, and cause menopausal symptoms (1). Some of the menopausal symptoms include vasomotor changes, palpitations, anxiety and insomnia, osteoporosis, cardiovascular disease, changes in the genital and urinary system, and depression. The most common symptoms include hot flashes and night sweats that occur at night mostly (2). Naturally, hot flashes and sweating have no risk for health, but cause some problems that affect daily functions (3). Hot flashes are the main symptom of the climacteric period that postmenopausal women have, in different degrees (2). Most women are looking for the way to reduce or treat hot flashes, especially if these symptoms disturb their daily performance (4). The most effective method to reduce or treat hot flashes is hormone replacement therapy (5–7); However due to side effects of this method such as heart attacks, hypertension, coronary artery disease, stroke, thromboembolism and breast cancer, the use of hormone therapy is very limited (8–10). During the past decade, increasing the use of alternative therapies such as nutrition, exercise, relaxation, and complementary medicine has reduced the menopausal symptoms. One of the most common of these methods is the use of medicine herb (11). The World Health Organization (WHO) considered the complementary medicine as a method for prevention and improvement of menopausal symptoms (12). Due to menopause complications attributed to estrogen deficiency, it seems that the use of plant estrogens is effective on reducing some of the menopausal symptoms. Phytoestrogens are non-herbal plants steroid that has estrogenic properties (13). *Salvia* from the Lamiaceae family is one of these phytoestrogens used as a medicinal herb in Iranian medicine. This is a native plant of Mediterranean Europe that is cultivated in East Azerbaijan and gardens in some other areas. Recent research has emphasized on some of the properties of this plant including antibiotics, anti-spasmodic, anti-anxiety, anti-fungal, anti-toxic, hypo-glycemic, estrogenic properties, and treatment of menopause pain (4, 14, 15). In Europe, *Saliva* has been used to reduce sweating, and sweating due to mainly menopause and hot flashes traditionally, and its use as an antiperspirant has a long history (4). It was showed that *Saliva officinalis* has significantly reduced severity and duration of pain in girls with primary dysmenorrhea (16).

Considering previous documents about the effectiveness of this plant on the reduction of hot flashes in traditional medicine in Iran and also the world, the aim of this study was to investigate the effect of the sage extract on the menopausal symptoms.

## 2. Materials and Methods

**Figure 1:**
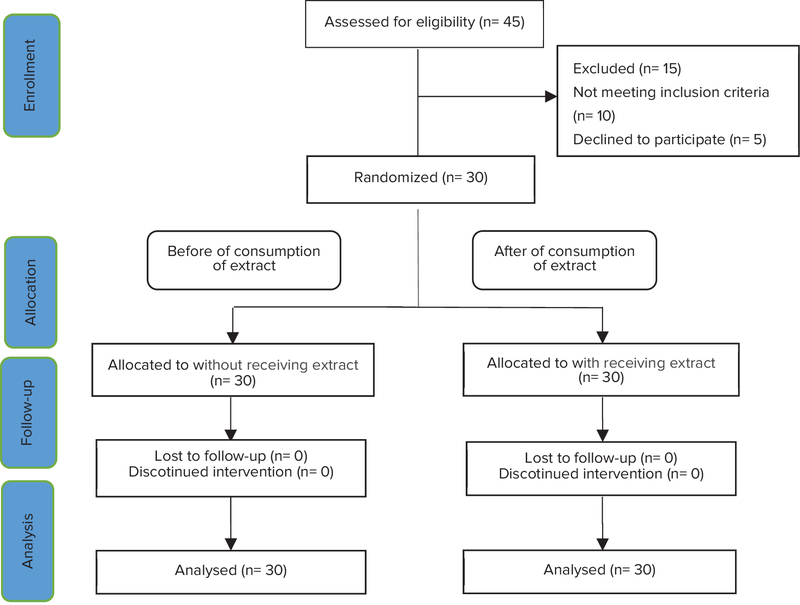
Consort flow diagram to select participants

**Figure 2:**
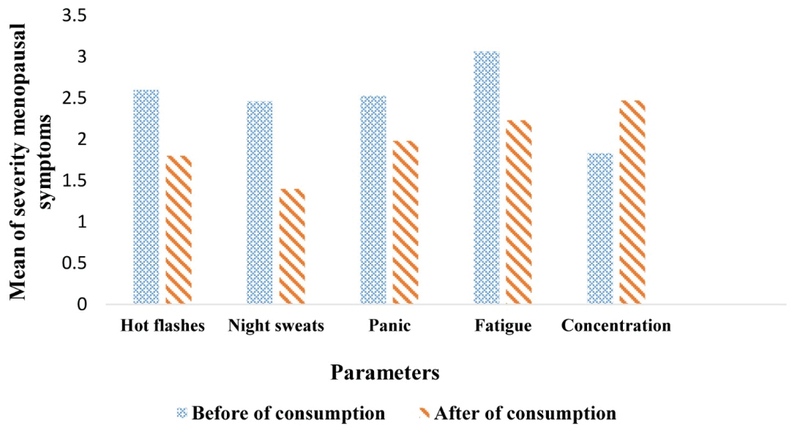
Mean of the severity of menopausal symptoms before and after the consumption of *Saliva* tablet

This interventional clinical trial (before and after study) was performed on 30 postmenopausal women referred to healthcare centers of Darab, Iran in the 2017 (Figure 1). Data were collected using a questionnaire including demographic information and the Menopause Rating Scale (prepared by the researcher. The demographic information questionnaire included age, the age at the time of last menstruation, employment status, economic status, and body mass index. Participants completed the MRS twice. All subjects who had experienced varying degrees of the hot flashes entered the study and were excluded from the study in case of the consumption of hormonal medications, underlying diseases, or sensitization to the herbal plant (Figure 2). After obtaining the informed consent from all participants, they filled the severity of menopausal symptoms before drug consumption in the first week. Then, they received one oral tablet of saligol (Gol Daru Company) for four weeks daily. Each tablet contained 100 mg dry extract of sage. At the end of the week, they completed the questionnaire again. The severity of menopausal symptoms was determined based on the theory proposed by the World Food and Drug Administration, the conversion of the qualitative variable intensity to a quantitative; 1 for never, 2 for a few, 3 for mild, and 4 for intense was considered. At the end of the fourth week, information forms were collected from all participants, again.

### Ethical consideration

Oral consent was obtained from all participants. The study proposal was approved by the ethics committee of Payame Noor University (code: IR.PNU.REC.1397.024).

### Statistical analysis 

Data were analyzed using Wilcoxon test by SPSS software (Statistical Package for the Social Sciences, version 17.0, SPSS Inc, Chicago, Illinois, USA). p≤ 0.05 was considered as significant.

## 3. Results

The mean±SD age of participants was 52.6±0.643, mean of age at the time of last menstruation 51.4±0.706, mean children's 4.4±0.207, and the mean of body mass index was 25.1±0.486. Other demographic factors were hemogenous between the women. The results showed that the mean of the severity of some of the postmenopausal symptoms including hot flashes, night sweats, panic, fatigue, and concentration had significant differences after the consumption of *Saliva* tablet compare to before consumption (p≤ 0.05). The findings demonstrated that there were no significant differences in other symptoms including heart rate, breathing, sadness, headache, sexual desire, itching, crying and interest in doing things (Table I & Figure 2).

**Table 1:** Mean±S.E of postmenopausal symptoms before and after of saliva tablet consumption


**Levels**	**Before of consumption**	**After of consumption**	**P value**
**Parameters**		
Hot flashes	2.6 ± 0.177	1.8 ± 0.155	0.00
Night sweat	2.46 ± 0.178	1.4 ± 0.123	0.00
Panic	2.53 ± 0.184	1.98 ± 0.167	0.02
Fatigue	3.07 ± 0.155	2.23 ± 0.136	0.02
Concentration	1.83 ± 0.145	2.47 ± 0.184	0.00
Heart rate	1.9 ± 0.175	1.73 ± 0.126	0.06
Breathing	1.1 ± 0.1	1.13 ± 1.04	0.33
Sadness	2.8 ± 0.169	2.8 ± 0.162	0.16
Headache	1.9 ± 0.154	1.87 ± 0.133	P= 0.71
Sexual desire	1.63 ± 0.162	1.67 ± 0.146	P= 0.57
Itching	1.53 ± 0.15	1.5 ± 0.142	P= 0.33
Crying	3.1 ± 0.194	3 ± 0.192	P= 0.18
Interest in doing things	1.8 ± 0.2	1.87 ± 0.196	P= 0.42
Wilcoxon test			

## 4. Discussion

In this study, the effect of *Salvia *officinalis on the menopausal symptoms in postmenopausal women was investigated and findings show that the use of the extract of the sage can reduce some menopausal symptoms including hot flashes, night sweats, panic, fatigue, and concentration. Many studies have been carried out in the field of the effects of phytoestrogens plants on the treatment of hot flashes in menopause, including the effect of flax seed on the hot flashes (17), licorice root extract on reducing the intensity and number of hot flashes in postmenopausal women (18) and Glycine* max* on reducing the frequency of hot flashes (19). Also, the sedative effect of the aqueous and alcoholic extract of *Saliva* has been proved (20, 21). Another study indicated that there is a significant positive effect of these phytoestrogens on the reduction of hot flashes in postmenopausal women (22). The sage extract and placebo were effective on reducing the frequency of hot flashes, but the sage was more effective (22). Also, it was seen that the combination of extract of the herb with alfalfa hay after three months of continuous use leads to the reduction of menopausal symptoms, the phytoestrogens in these plants may be due to anti-dopaminergic effects on the central nervous system and that neurotransmitters reduce these symptoms (23). These findings are consistent with the present research.

In a study in order to determine the efficacy of the sage extract on the hot flashes in postmenopausal women, Boomer and colleagues concluded that the use of herbal extracts significantly reduced the severity and number of hot flashes in postmenopausal women (24). Studies showed that treatment of alfalfa and sage had a significant increase in estradiol level than control group. Histological studies showed a significant increase in the number of ovarian follicles and increasing the diameter of the endometrial glands (25). In another study, the effect of sage and placebo checked out on the frequency of hot flashes in postmenopausal women, and results revealed that there were no significant differences between sage and placebo groups with respect to the reduction of frequency, duration, and severity of hot flashes in the sage group (26). Also, the efficacy of sage was evaluated in controlling hot flashes in patients with prostate cancer; the findings showed that the number and severity of the symptoms were significantly reduced (27). In postmenopausal women treated with sage extract, hot flashes and night sweats had significant decrease compared to the control group, but there were no significant differences in estradiol level between the two groups (28).

## 5. Conclusion

The findings of the this study showed that tablet of sage reduced the severity hot flashes, night sweats, panic, fastigue and increased concentraion. So it can be used saliva for improved the Menopausal symptoms in menopausal women.

##  Conflict of Interest 

The authors declare that there is no conflict of interest in the current study.
